# Intraspecific Chloroplast Genome Variation and Domestication Origins of Major Cultivars of *Styphnolobium japonicum*

**DOI:** 10.3390/genes14061156

**Published:** 2023-05-26

**Authors:** Zhiqiang Mu, Yu Zhang, Bin Zhang, Yueqin Cheng, Fude Shang, Hongwei Wang

**Affiliations:** 1 College of Landscape Architecture and Art, Henan Agricultural University, Zhengzhou 450002, China; 2 College of Plant Protection, Henan Agricultural University, Zhengzhou 450002, China; chengyq126@126.com; 3 College of Life Science, Henan Agricultural University, Zhengzhou 450002, China

**Keywords:** *Styphnolobium japonicum*, chloroplast genome, genetic diversity, highly variable region, domestication origin

## Abstract

*Styphnolobium japonicum* is a significant resource of ornamental and medicinal plants. In this study, we employed high-throughput sequencing to assemble nine chloroplast genomes of *S. japonicum*. We compared and reconstructed the phylogenetic relationships of these genomes, along with three publicly available chloroplast genomes. Our results showed that the length of the 12 *S. japonicum* chloroplast genomes ranged from 158,613 bp to 158,837 bp, all containing 129 unique functional genes. The genetic diversity within *S. japonicum* chloroplast genomes was relatively low, with π = 0.00029, Theta-W = 0.00028, and an indel frequency of 0.62 indels/1 kb. Among the four regions, the SSC region exhibited the highest genetic diversity and indel frequency, while the IR region had the lowest. Non-coding regions displayed greater genetic variation compared to coding regions, with a few highly variable regions identified. The phylogenetic tree constructed revealed that the major cultivars of *S. japonicum* originated from two genetic ‘sources. *S. japonicum* ‘JinhuaiJ2’ had an independent origin and showed close relatedness to *S. japonicum* var. *violacea*, *S. japonicum* var. *japonicum*, and *S. japonicum* f. *oligophylla*. On the other hand, other major cultivars shared a common genetic origin and were closely related to *S. japonicum* f. *pendula*. This study highlights the variability of chloroplast genomes within *S. japonicum* and provides insights into the genetic origins of major cultivars and their relationships with different varieties and forma.

## 1. Introduction

*S. japonicum*, also known as the pagoda tree, belongs to the Leguminosae family, subfamily Faboideae, tribe *Sophoreae*, and genus *Styphnolobium*. It is originally from China and has been introduced to several countries in Europe and America. The pagoda tree has a long history of medicinal use, and its roots, branches, bark, leaves, flowers, and fruits are recorded or described as herbal medicine in traditional Chinese pharmacopoeias such as the Compendium of Materia Medica and the Chinese Pharmacopoeia [1,2,3]. The unopened flower (Huaimi) of the pagoda tree can also be used for the development of functional food products [4,5]. Modern research has shown that the flowers or Huaimis of the pagoda tree contain flavonoids such as rutin, quercetin, kaempferol, and their glycosides [6,7]. In addition to these components, the fruit of the pagoda tree also contains isoflavones [8]. The pagoda tree is not only used for medicinal purposes but also as a long-lived tree species that exhibits rich morphological variation in its tree form, branches, leaves, and flowers. It is often cultivated as an ornamental plant in gardens and landscaping [9,10]. According to the Flora of China, there are three varieties of pagoda tree (*S. japonicum* var. *pubescens*, *S. japonicum* var. *violacea*, and *S. japonicum* var. *vestita*), two forma (*S. japonicum* f. *oligophylla* and *S. japonicum* f. *pendula*), and multiple cultivars.

Most previous studies have focused on exploring the relationship between *S. japonicum* and its closely related species [11,12,13,14,15,16]. Based on the determination of peroxidase (POD) and esterase (EST) in the *Styphnolobium* plant, Li Xiaolin et al. believed that *S. japonicum* is more closely related to *S. japonicum* f. *pendula* and further away from species such as *Robinia pseudoacacia* ‘decaisneana’ and *Cladrastis sinensis* Hemsl, by comparing and analyzing the isozyme spectra and combining the plant’s morphological characteristics [17]. Heenan et al. [18] conducted a phylogenetic analysis of the position of *S. japonicum* among the *Styphnolobium* genus based on rbcL, ITS sequences, and morphological characteristics and determined its position within the genus. Lu et al. [19] and Shi et al. [20] separately sequenced and assembled the chloroplast genomes of *S. japonicum* var. *violacea* and *S. japonicum* ‘Jinhuai J2’, and then constructed a phylogenetic tree based on chloroplast genome variations to reveal their respective positions in the Fabaceae family. In recent years, Sun et al. [21] used SRAP markers to amplify and detect 34 samples of *S. japonicum* and explored the genetic relationships between these samples based on the genetic similarity coefficient and UPGMA clustering. However, this study did not include genetic information from *S. japonicum* varieties and forma and only used samples from some cultivars, most of which were clone plants. Therefore, our understanding of the genetic information of *S. japonicum* varieties and forma is still limited, which makes it difficult to clarify the relationships between these cultivars and forma. These limitations in knowledge have also affected the cultivation and further development and utilization of high-quality *S. japonicum* varieties.

Chloroplasts are important organelles in plant cells, which not only have functions such as photosynthesis, carbon assimilation, fatty acid, and amino acid biosynthesis but also play roles in other physiological and developmental processes [22,23]. In most angiosperms, chloroplast DNA is maternally inherited and has many advantages in molecular biology research. The chloroplast genome is relatively small (107 kb–218 kb), with a high copy number within cells, and is easy to sequence. It is also rarely affected by paralogous genes. The structure of the chloroplast genome is conserved, the number of genes is stable, and there is good collinearity, making it suitable for sequence alignment analysis. In addition, the chloroplast genome has an evolutionary rate between that of the nuclear genome and mitochondrial genome, and the coding and non-coding regions have different evolutionary rates, which can be used to analyze the phylogenetic relationships of different taxa [24]. Therefore, the chloroplast genome has been widely used in studies of systematics, species identification, and phylogeography [25,26,27]. Additionally, chloroplast genes can also be used for crop improvement. In 1988, Boynton et al. [28] used gene gun technology to achieve chloroplast genetic transformation in *Chlamydomonas reinhardtii*, demonstrating the feasibility of cpDNA transformation. Olejniczak et al. [29] and Bock et al. [30] have explored the prospects of chloroplast transformation technology in improving crop characteristics (such as resistance to pests and diseases and herbicide tolerance), metabolic engineering, and molecular agriculture. This study employed high-throughput sequencing to assemble the chloroplast genomes of nine distinct phenotypes of *S. japonicum*. Subsequently, this study aimed to investigate various scientific issues, including the following: (1) the structure, genome composition, and genetic variation of the chloroplast genome within *S. japonicum*; (2) the identification of highly variable sites in the chloroplast genome of *S. japonicum*; (3) clarification of the phylogenetic relationships among *S. japonicum* varieties, forms, and major cultivars. These results provide a theoretical basis for molecular identification, plant genetic improvement, and selection of excellent varieties of *S. japonicum*.

## 2. Materials and Methods

### 2.1. Sample Collection

**Figure 1:**
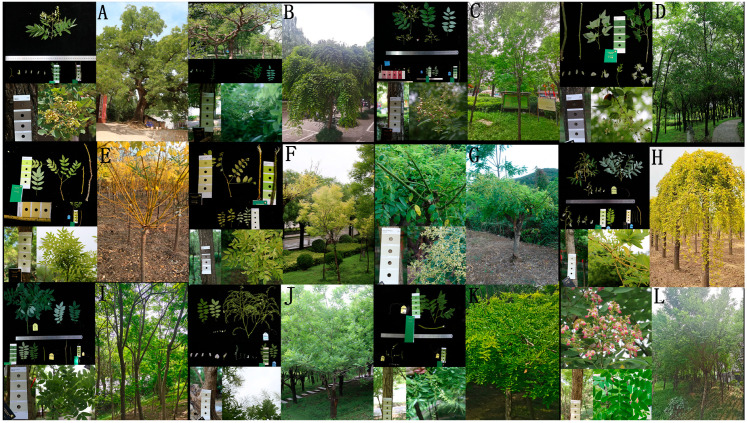
Morphological characteristics of twelve *Styphnolobium japonicum* samples. (**A**) *S. japonicum* var. *Japonicum*; (**B**) *S. japonicum* f. *pendula*; (**C**) *S. japonicum* var. *Violacea*; (**D**) *S. japonicum* f. *oligophylla*; (**E**) *S. Japonicum* ‘Winter Gold’; (**F**) *S. japonicum* ‘Flavi-rameus’; (**G**) *S. japonicum* ‘JinhuaiJ2’; (**H**) *S. japonicum* ‘Jinyechui’; (**I**) *S. japonicum* ‘Susheng’; (**J**) *S. japonicum* ‘Shuangjimi’; (**K**) *S. japonicum* ‘Pingding’; (**L**) *S. japonicum* ‘Liaohong’.

Leaf samples were collected from the Chinese Huaiyuan garden in Shenqiu County, Zhoukou City, Henan Province, China (115°6′06.30″ E, 33°24′58.90″ N). The samples were obtained from nine variations and cultivars of *S. japonicum*, including *S. japonicum* f. *oligophylla* (Five-leaf Scholar Tree) with 1–2 pairs of leaflets growing at the apex of the leaf axis, forming a palm-shaped structure as a whole. *S. japonicum* f. *pendula* (Dragon Claw Scholar Tree) has branches that bend, spiral, and droop downward in all directions. *S. japonicum* ‘Winter Gold’ exhibits golden yellow branches, while *S. japonicum* ‘Flavi-rameus’ showcases golden yellow leaflets. *S. japonicum* ‘Liaohong’ features light pink banner petals and pale lilac wing and keel petals. *S. japonicum* ‘Shuangjimi’ is known for blooming twice a year. *S. japonicum* ‘Jinyechui’ has golden yellow leaflet and drooping downward branches. *S. japonicum* ‘Pingding’ has a flat crown and an overall umbrella-shaped tree form, while *S. japonicum* ‘Susheng’ exhibits fast growth (Figure 1). Fresh and healthy leaf samples were quickly dried using silica gel for preservation at room temperature until DNA extraction.

### 2.2. Plant Total DNA Extraction, Genome Sequencing, and Assembly

The improved CTAB method was used for total genomic DNA extraction, and the purity and concentration of DNA were assessed via 1% agarose gel electrophoresis and Nanodrop (Thermo Fisher Scientific 5225 Verona Rd. Madison, WI53711 Assembled in USA). After the DNA samples passed the quality control, library construction, and library quality testing were carried out. Finally, the Illumina high-throughput sequencing platform NovaSeq 6000 (Novogene Company Limited, Beijing, China) was used for paired-end (2 × 150 bp) sequencing of DNA libraries. Each sample obtained at least 6 G of raw sequence data, which was then filtered to obtain clean data. Using the reference sequence of the chloroplast genome of *S. japonicum* var. *japonicum* (MG784459), the chloroplast genomes of various samples were assembled using spades v3.15.3 software (Linux system) [31]. The optimal assembly result was obtained by adjusting the K-mer value. The chloroplast genome sequences were annotated using the online website CPGAVAS2 (http://47.96.249.172:16019/analyzer/home; last accessed 30 October 2022) and then manually corrected using Geneious 9.0 software [32]. The other three chloroplast genome sequences of *S. japonicum* were obtained from the NCBI database, including *S. japonicum* var. *Japonicum*, *S. Japonicum* var. *Violacea*, and *S. japonicum* ‘Jinhuai J2’. To avoid sequence differences caused by human factors and affecting subsequent analysis, these chloroplast genomes were re-annotated and adjusted. Finally, the 9 newly assembled and annotated complete chloroplast genome sequences in this study were uploaded to the NCBI database with the following accession numbers: ON571618, ON571617, ON571614, N571615, ON571620, ON571619, ON571616, ON571621, and ON553276. The physical map of the chloroplast genome was drawn using the online website OGDRAW version 1.3.1 (https://chlorobox.mpimp-golm.mpg.de/OGDraw.html; last accessed 15 November 2022) [33].

### 2.3. Chloroplast Genome Comparative Analysis

To detect the overall similarity of chloroplast genomes within *S. japonicum*, we used the online website mVISTA (https://genome.lbl.gov/vista/mvista/submit.shtml; last accessed 25 November 2022) and selected the Shuffle-LAGAN mode to compare the complete chloroplast genome sequences of 12 *S. japonicum* individuals, with a similarity range of 50–100. We used Geneious9.0 software for performing MAFFT (multiple sequence alignment program) alignment on the 12 chloroplast genome sequences. We also conducted collinearity analysis of the *S. japonicum* chloroplast genome using Mauve aligner. Then, we partitioned the aligned chloroplast genomes into two categories, one based on structure, which divided the chloroplast genome into one large single-copy region (LSC), one small single-copy region (SSC), and two inverted repeat regions (IRa, IRb), and the other based on coding or non-coding regions. Next, we used DnaSP6 v6.12.03 [34] software to detect SNP sites in the chloroplast genome and in each partition and calculate their genetic diversity (Pi value and Theta-W). The two inverted repeat regions of the chloroplast genome form four boundary regions between the large and small single-copy regions, which are LSC/IRb (JLB), IRb/SSC (JSB), SSC/IRa (JSA), and IRa/LSC (JLA). We used the IRscope script (Linux) to visualize the expansion and contraction of the four boundary regions of the *S. japonicum* chloroplast genome [35]. We used the online website MISA (https://webblast.ipk-gatersleben.de/misa/index.php?action=1; last accessed 27 November 2022) to identify microsatellite sequences (SSRs) in each chloroplast genome [36], and the minimum repeat number of various types of SSR repeat motifs was set to 10 for mononucleotide repeats, 5 for dinucleotide repeats, 4 for trinucleotide repeats, 3 for tetranucleotide repeats, and 3 for both pentanucleotide and hexanucleotide repeats.

### 2.4. Phylogenetic Analysis

In order to explore the phylogenetic relationships among different varieties, forma, and cultivars of *S. japonicum*, we used *Maackia amurensis*, *Cladrastis yungchunii*, and *R. pseudoacacia* as outgroups (genomic data obtained from NCBI). We aligned 15 chloroplast genomes via MAFFT in phylosuite v1.2.2 software [37] and used ModelFinder to detect the best nucleotide substitution models for Maximum likelihood (ML) and Bayesian inference (BI) methods. We constructed the phylogenetic tree using both ML and BI methods. The BI method was based on the Markov chain Monte Carlo (MCMC) algorithm, running for 1,000,000 generations with a tree sampled every 1000 generations. The first 25% of sampled trees were discarded as burn-in, and the remaining trees were used to construct a consensus tree. The ML method was performed using IQtree software [38], with the bootstrap method repeated 1000 times. Finally, the tree was optimized using the online iTOL website (https://itol.embl.de/; last accessed 29 November 2022).

## 3. Results

### 3.1. Characteristics of the Chloroplast Genome of S. japonicum

**Figure 2:**
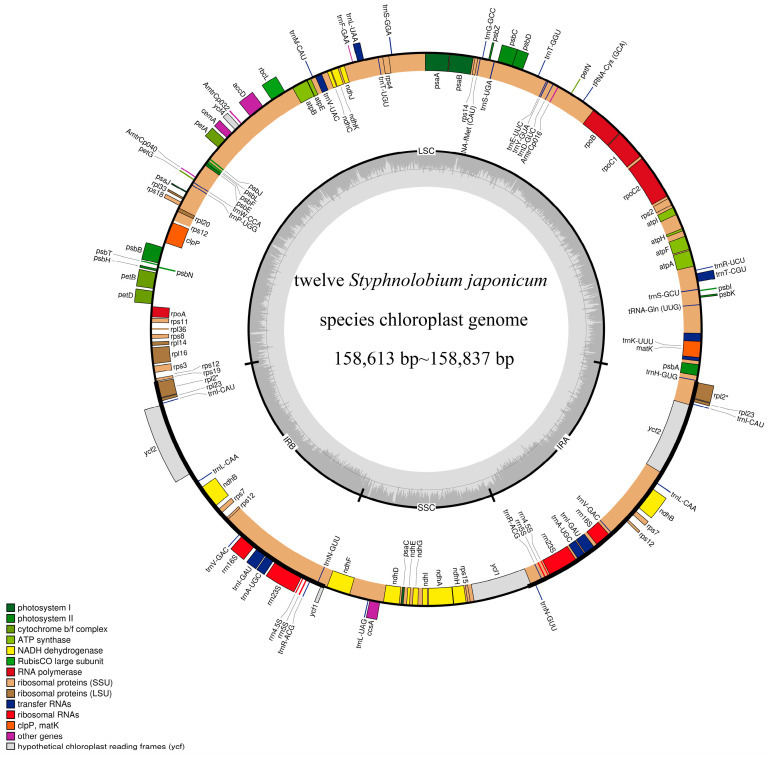
Map of the chloroplast genome of *S. japonicum*. Genes drawn inside the circle are transcribed clockwise, and those outside the circle are transcribed counterclockwise. The dashed gray area in the inner circle shows the percent GC content of the corresponding genes. LSC, SSC, and IR denote large single copy, small single copy, and inverted repeats, respectively. The circular map of the chloroplast genome was drawn using OGDRAW 1.3.1.

The size of the chloroplast genomes of the 12 *S. japonicum* samples after alignment was 159,102 bp, and the size of each chloroplast genome ranged from 158,613 bp to 158,837 bp (Figure 2 and Table 1). All chloroplast genomes showed a typical quadripartite structure, consisting of a large single-copy region (LSC; 88,868–88,977 bp), a small single-copy region (SSC; 19,034–19,073 bp), and two inverted repeat regions (IR; 50,678–50,796 bp). The GC content of the genome was almost identical (36.1%). In addition, the gene content and arrangement of the 12 *S. japonicum* chloroplast genomes were identical. They all contained 129 unique functional genes, including 83 protein-coding genes (PCGs), 38 tRNA genes, and 8 rRNA genes (Table 2). Based on the functions of the genes, the chloroplast genome-encoded genes can be divided into four categories: Photosynthesis, Self-replication, Other genes, and Genes of unknown function. Among these genes, 18 were repeat genes, each with 2 copies, including 7 protein-coding genes (*ycf2*, *ycf1*, *rps12*, *rps7*, *rpl23*, *rpl2*, and *ndhB*), 4 rRNA genes (*rrn23S*, *rrn16S*, *rrn5S*, and *rrn4.5S*), and 7 tRNA genes (*trnA-UGC*, *trnI-CAU*, *trnI-GAU*, *trnL-CAA*, *trnN-GUU*, *trnR-ACG*, and *trnV-GAC*).

### 3.2. Chloroplast Genome Comparative Analysis

**Figure 3:**
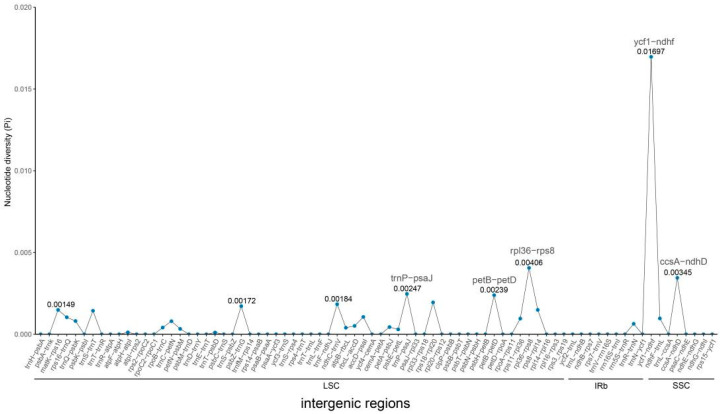
Nucleotide diversity of noncoding regions among twelve *S. japonicum* samples. X-axis: the position in the genome (noncoding region); Y-axis: Pi value. Pi, nucleotide polymorphism.

**Figure 4:**
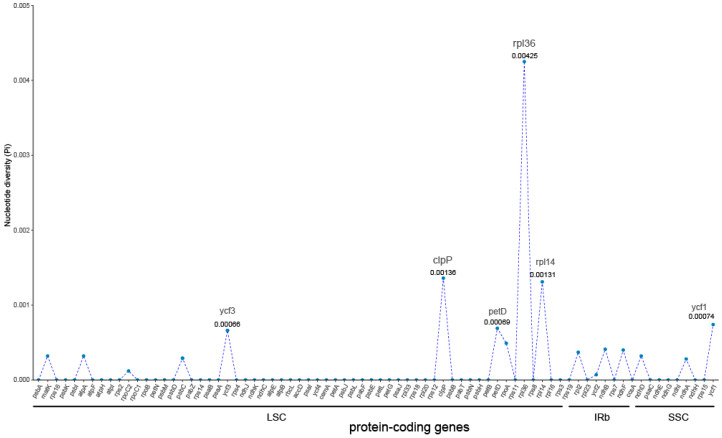
Nucleotide diversity of coding regions among twelve *S. japonicum* samples. X-axis: the position in the genome (coding region); Y-axis: Pi value. Pi, nucleotide polymorphism.

The comparative analysis of the chloroplast genome of *S. japonicum* shows that its composition and structure are highly conserved. Variations are mainly concentrated in non-coding regions while coding regions show higher conservation. Some nucleotide high variability regions, such as *ycf1-ndhF*, *rpl36-rps8*, and *petB-petD*, exhibit polymorphic specificity peaks among different species (Figure S1). Moreover, *S. japonicum* f. *oligophylla* and *S. japonicum* var. *violacea* have the least variable sites with the original variety of *S. japonicum*, indicating a high degree of similarity. Further analysis revealed 132 SNP loci in the 12 chloroplast genomes of *S. japonicum*, with a total genetic polymorphism of Pi = 0.00029 and Theta-W = 0.00028 (Table 3). Among them, the SSC region has the highest polymorphism, detecting 38 SNPs, with Pi and Theta-W values of 0.00085 and 0.00066, respectively. The polymorphism of the LSC region is significantly lower than that of the SSC region, with a total of 59 SNPs and Pi and Theta-W values of 0.00026 and 0.00022, respectively. The genetic polymorphism of the IR region is the lowest, with Pi and Theta-W values of 0.00014 and 0.00024, respectively, which is only half of that of the LSC region. The total length of the non-coding region of the chloroplast genome in *S. japonicum* is 81,133 bp, with 87 SNPs and the same Pi and Theta-W values of 0.00043. The total length of the coding region is 78,072 bp, with 45 SNPs and Pi and Theta-W values of 0.00019 and 0.00019, respectively. We further detected genetic variation in all non-coding and coding regions separately and found that 26 non-coding regions and 17 coding regions had nucleotide mutations (Figure 3 and Figure 4). These non-coding regions with nucleotide variation have only four located in the SSC and IR regions, and the remaining 22 are in the LSC region, with an average genetic polymorphism of 0.00185. Among them, the ycf1-ndhf non-coding region in the SSC region has the highest genetic variation, with a polymorphism level far higher than that of other non-coding regions. The Pi value of this region is as high as 0.01697, and the Theta-W value is 0.01325. The genetic polymorphism Pi values of other non-coding regions are all less than 0.005, less than one-third of that of *ycf1-ndhf*. The non-coding region with higher genetic polymorphism is *rpl36-rps8*, with Pi and Theta-W values of 0.00406 and 0.00301, respectively, followed by *ccsA-ndhD*, with a Pi value of 0.00345 and Theta-W value of 0.00236. The other two high variability regions are *trnP-psaJ* (Pi = 0.00247) and *petB-petD* (Pi = 0.00239), which have similar genetic polymorphisms. In addition, there are 13 other non-coding regions with genetic diversity Pi values greater than 0.001. The genetic polymorphism in the coding region is significantly lower than that in the non-coding region, with an average genetic polymorphism of only 0.00073, and only three coding regions have a genetic polymorphism Pi > 0.001. The rpl36 genetic variation located in the LSC region is the highest, with Pi = 0.00425 and Theta-W = 0.00290, followed by *clpP* (Pi = 0.00360, Theta-W = 0.00224) and *rpl14* (Pi = 0.00131, Theta-W = 0.00090).

Insertions and deletions (indels) are another common type of genetic variation. After sequence alignment of the chloroplast genomes of 12 *S. japonicum*, 98 indels were detected with a frequency of 0.62 indels/1 kb. Among the four regions of the chloroplast genome, SSC had the highest frequency of indels, which was 1 indel/1 kb. The indel frequency in the LSC region was slightly lower, at 0.8 indels/1 kb, while the indel frequency in the IR region was the lowest, at only 0.16 indels/1 kb. At the same time, indels mainly occurred in the non-coding region, where the frequency of indels was 1.2 indels/1 kb, and only two indels appeared in the coding region. In the *S. japonicum* chloroplast genome, the longest indels were 54 bp and 38 bp, while most of the other indels were 1 bp (single nucleotide). The two longest indels appeared in the non-coding region of the IR region and LSC region, respectively, located in the intron of tRNA-UGC and the intergenic spacer between matk and *rps16*. The indels that appeared in the protein-coding region were the *matk* and *rps19* genes, with lengths of 6 bp and 1 bp, respectively.

### 3.3. Chloroplast Genome Collinearity and Partition Boundary Analysis

**Figure 5:**
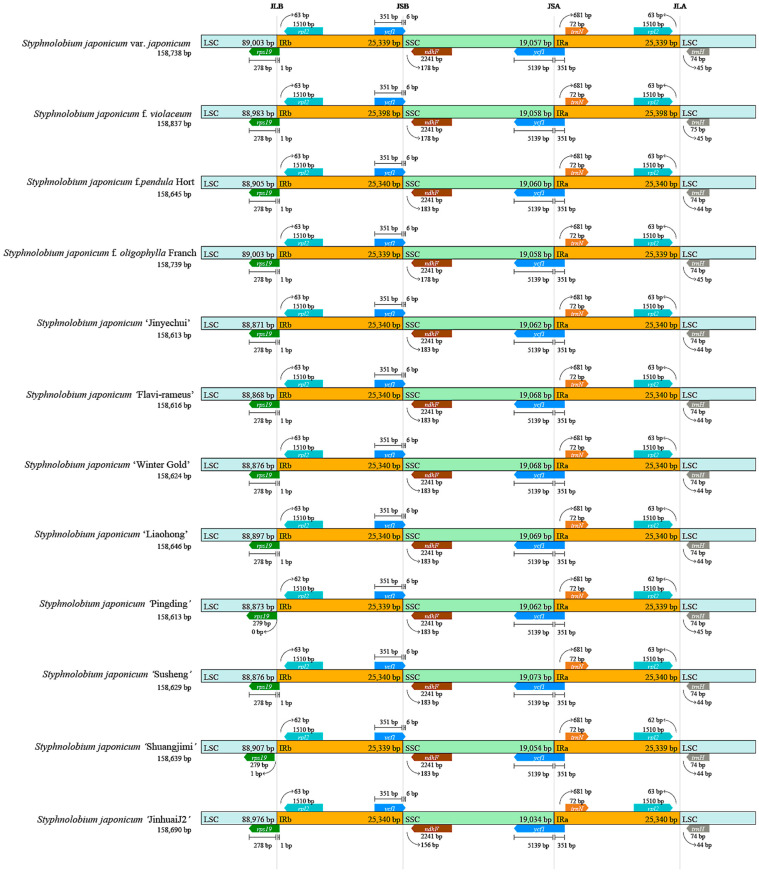
Comparison of the junction between large single copy (LSC), small single copy (SSC), and inverted repeat (IRs) regions of chloroplast genome among twelve *S. japonicum* samples.

The collinearity analysis of the chloroplast genome among different varieties of *S. japonicum* showed that, apart from variations in base pair lengths, the gene positions and directions remained consistent, and there was no occurrence of rearrangement or inversion (Figure S2). The composition and structure of the four partition boundaries in the chloroplast genome of *S. japonicum* were also relatively stable (Figure 5).

In JLB, the distance of *rpl19* from the boundary was 0 bp and 1 bp for *S. japonicum* ‘Pingding’ and *S. japonicum* ‘Shuangjimi’, respectively. In other chloroplast genomes, *rpl19* was distributed across this boundary, with its 278 bp located in LSC and only 1 bp in IRb. Another gene, *rpl2*, was located entirely in IRb, and its distance from the boundary was 62–63 bp. *ycf1* crossed the JSB boundary and was distributed in IRb and SSC with stable lengths of 351 bp and 6 bp, respectively, while another gene, *ndhF*, which was entirely distributed in SSC, was located 156 bp–183 bp from the boundary. Similarly, another copy of *ycf1* crossed the JSA boundary and was distributed in SSC and IRa with stable lengths of 5139 bp and 351 bp, respectively. Moreover, the *trnN* located at this boundary was all within IRa and was 681 bp from the boundary. No gene crossed the JLA boundary, and like the JLB boundary, *rpl2* distributed in the IRa region was 62–63 bp from the boundary, while *trnH* located in LSC was 44–45 bp from the boundary.

### 3.4. Chloroplast Genome Repetitive Sequence Analysis

**Figure 6:**
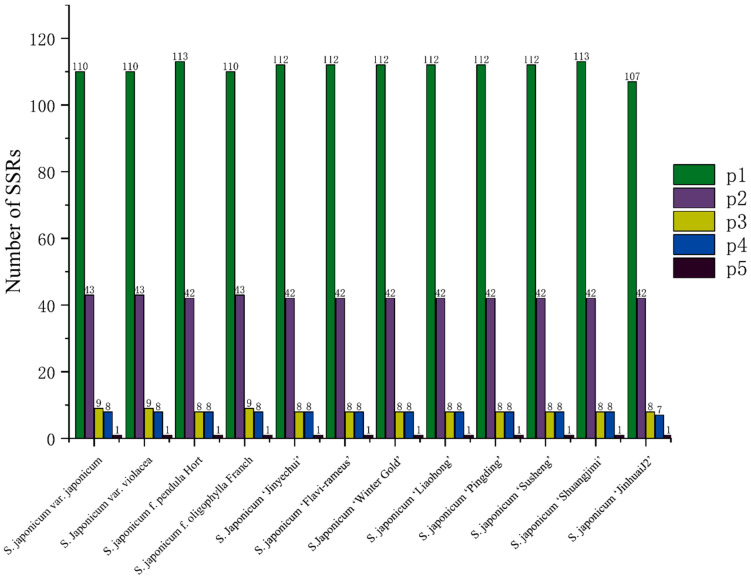
The number of various types of SSRs in twelve *S. japonicum* chloroplast genomes. p1: Mono. p2: Di. p3: Tri. p4: Tetra. p5: Penta.

**Figure 7:**
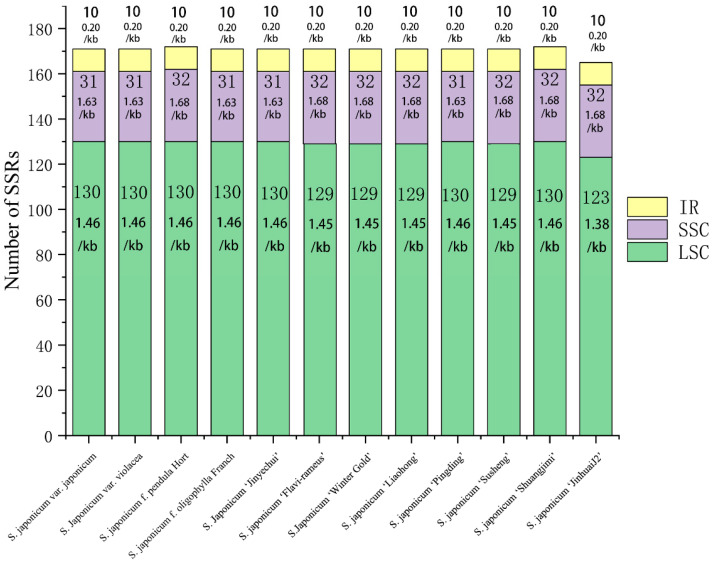
Distribution of SSRs in twelve *S. japonicum* chloroplast genomes.

Five types of SSR were detected in the *S. japonicum* chloroplast genome (Figure 6), with repeat units of mono-, di-, tri-, tetra-, and penta-nucleotides. The number of SSRs of different types varied greatly, with SSRs containing mono-nucleotide repeat units being the most abundant. The number of these SSRs in a single chloroplast genome ranged from 107 to 113, accounting for 64.3% to 65.7% of the total SSRs, with an average of 65.2%. SSRs containing di-nucleotide repeat units were also common, accounting for 24.4% to 25.5% of the total SSRs, with an average of 24.8%. SSRs containing tri- and tetra-nucleotide repeat units were relatively rare, with occurrence rates of 4.8% and 4.6%, respectively. SSRs containing penta-nucleotide repeat units were the least common, with only one per chloroplast genome and an occurrence rate of only 0.6%. The frequency of SSRs in chloroplast genomes of different *S. japonicum* cultivars, varieties, and ecotypes was very similar, except for *S. japonicum* ‘JinhuaiJ2’, which had a frequency of 1.04 SSR/kb, while all other chloroplast genomes had a frequency of 1.08 SSR/kb (Figure 7). These SSR loci were unevenly distributed in the chloroplast genome but followed the same pattern in different chloroplast genomes, with more abundant SSRs in the single-copy region and significantly fewer SSRs in the inverted repeat region. The frequency of SSRs was highest in the SSC region, with 1.63–1.68 SSR/kb and an average of 1.66 SSR/kb. The frequency of SSRs in the LSC region was slightly lower than that in the SSC region, with 1.38–1.46 SSR/kb and an average of 1.45 SSR/kb. All chloroplast genomes had only 10 SSRs in the IR region, with a frequency of 0.20 SSR/kb, significantly lower than that in the SSC and LSC regions.

### 3.5. Phylogenetic Analysis of the S. japonicum Species

**Figure 8:**
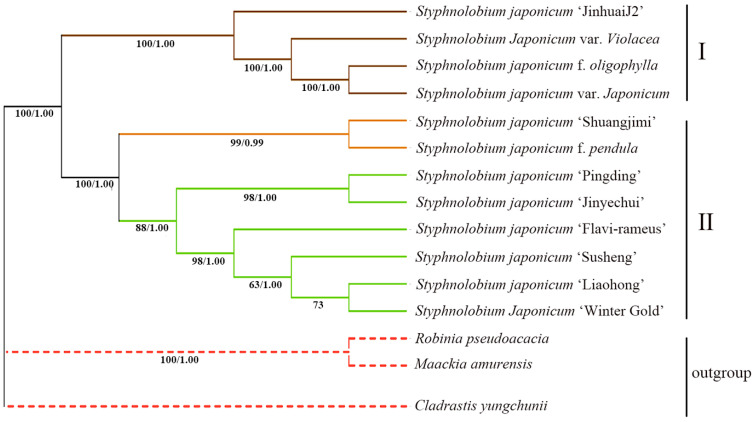
Phylogenetic tree based on whole chloroplast genome sequence. The numbers to the left of the slashes on the branches show the bootstrap values obtained by maximum likelihood analyses, and those to the right show the posterior probabilities according to Bayesian inference.

Phylogenetic trees for *S. japonicum* samples were constructed using both ML and BI methods based on complete chloroplast genome sequences. *M. amurensis*, *Cladrastis yungchunii*, and *R. pseudoacacia* were used as outgroups. Both ML and BI trees exhibited consistent topologies, with maximum bootstrap support and posterior probabilities of 100% for all major branches (Figure 8). The phylogenetic trees divided the *S. japonicum* samples into two main clades, a small clade (I) consisting of two varieties (*S. japonicum* var. *violacea*, *S. japonicum* var. *japonicum*), one forma (*S. japonicum* f. *oligophylla*), and one cultivar (*S. japonicum* ‘JinhuaiJ2’), and a large clade (II) consisting of seven cultivars and one forma. All nodes within the small clade also had maximum support (100%/1.00). *S. japonicum* var. *japonicum* and *S. japonicum* f. *oligophylla* were the most closely related within the small clade, forming the sister group to *S. japonicum* var. *violacea*. Within the large clade, *S. japonicum* ‘Shuangjimi’ and *S. japonicum* f. *pendula* formed a clade that was sister to the other six cultivars. Within the combination clade of the other six cultivars, *S. japonicum* ‘Pingding’ and *S. japonicum* ‘Jinyechui’ formed a clade that was sister to the clade consisting of the other four cultivars. The support for the nodes within the combination clade of the remaining four cultivars was relatively low, especially for *S. japonicum* ‘Susheng’, *S. japonicum* ‘Liaohong’, and *S. japonicum* ‘Winter Gold’, whose relationships require further investigation.

## 4. Discussion

### 4.1. The Low Variation Level of the Chloroplast Genome in S. japonicum

The overall variation level of the chloroplast genome in *S. japonicum* is low, with a typical circular tetrad structure that is conserved in terms of genome length, structure, GC content, and genome composition. The IR region of the chloroplast genome is considered the most conservative region, but the sequence of the boundary region may expand outward or contract inward, leading to changes in the copy number of related genes or the production of pseudo-genes in the boundary region. This is a common phenomenon in chloroplast genome evolution and the main cause of its length variation [39,40]. The *rpl19*, *rpl2*, *ycf1*, *trnF*, *ycf1*, and *trnN* genes distributed at the boundary of the IR region within *S. japonicum* have not undergone expansion or contraction, except for the *rps19* gene in *S. japonicum* ‘Shuangjimi’ and *S. japonicum* ‘Pingding’. The genetic diversity level of the chloroplast genome in *S. japonicum* is also low. A total of 132 SNPs and 98 indels were detected in the 12 chloroplast genomes of *S. japonicum*, with a total genetic polymorphism Pi = 0.00029, Theta-W = 0.00028, and an InDel frequency of 0.62 InDels/1 kb. This variation level is lower than that of some other species, such as *Tagetes erecta* and *Ricinus communis*. Jiang et al. compared the chloroplast genome variation in *T. erecta* and identified 139 SNPs [41], although the chloroplast genome of this species (152 Kb) is smaller than that of *S. japonicum* (158 Kb). In multiple chloroplast genomes of *R. communis*, 162 SNPs and 92 InDels were detected, with frequencies of 0.99 and 0.56 per kb [42], respectively. In *Quercus acutissima*, although 332 single nucleotide variants (SNVs) were detected, the majority of these SNVs were InDels (255), with only 77 SNPs, significantly lower than the other species mentioned above [43].

### 4.2. Regional Differentiation of Genetic Polymorphisms in Chloroplast Genome of S. japonicum

The genetic variations in the chloroplast genome of plants exhibit a pattern of local concentration distribution, and there are significant differences in genetic polymorphism between different regions. In most plant families and genera, the single-copy regions of the chloroplast genome have higher genetic diversity than the inverted repeat regions [44,45]. For example, in 11 *Rubus* species (Rosaceae), Pi values vary greatly in the SSC and LSC regions, while the IR region is relatively conserved with an average value of 0.008 [46]. Pi values in different regions of *Calycophyllum Spruceanum* [47] also show that coding regions are more conserved than non-coding regions, and the SSC and LSC regions exhibit higher variation than the IR region. Similarly, a pattern of localized genetic variation also occurred within the chloroplast genome of individual species [48]. In the chloroplast genome of *S. japonicum*, Pi values in the SSC and LSC regions are 0.00085 and 0.00026, respectively, and their genetic polymorphisms are significantly higher than that in the IR region (0.00014), with the former being 6.5 and 2.1 times greater than the latter, respectively. The genetic diversity (π) in the IR region of *Euonymus maackii* is only 0.00086 [49], while the values in the SSC and LSC regions are as high as 0.00914 and 0.00562, respectively, which are 10.6 and 6.5 times greater than that in the IR region. Another pattern of regional variation within the chloroplast genome is that the genetic diversity in the non-coding regions is significantly higher than that in the coding regions. The Pi value of the non-coding region (0.00045) in the chloroplast genome of 12 *S. japonicum* individuals is 2.3 times higher than that in the coding region (0.00020), which is also similar to the ratio observed in E. maackii [49]. Su et al. calculated that the average genetic diversity (π) in the non-coding region of the chloroplast genome of *Aegilops tauschii* is 0.00133 [50], while that in the coding region is 0.000432, with a ratio of 3.1, slightly higher than that in *S. japonicum* and *A. tauschii*. The difference in genetic diversity between coding and non-coding regions also exists in families and genera above the species level. For example, in the *Paulownia* [51] genus, the genetic diversity of the non-coding region (Pi = 0.00102) is significantly higher than that of the coding region (Pi = 0.00033), with a ratio of 3.1, which is the same as that observed within *A. tauschii*. The lower genetic diversity in the coding regions of the chloroplast genome of plants may be due to functional constraints, which make these regions more conserved during evolution.

The genetic differences between coding and non-coding regions of chloroplast genomes are significant, and those non-coding and coding regions with large genetic variations are often referred to as hotspots of variation [52]. Some hotspot regions, such as *rbcL*, *nahF*, and *matK*, exhibit rich variations in many taxa and are commonly used as DNA barcodes for species identification [53,54]. However, the most highly variable regions are inconsistent across different taxa. The five non-coding hotspot regions with the highest variations in the chloroplast genome of *S. japonicum* are *ycf1-ndhf*, *rpl36-rps8*, *ccsA-ndhD*, *trnP-psaJ*, and *petB-petD*, and the three coding hotspot regions are *rpl36*, *clpP*, and *rpl14*. Arabidopsis thaliana has two significant peaks of nucleotide diversity, with *trnP-psaJ* being the same as in *S. japonicum* [55]. However, in *A. tauschii*, except for *ccsA-ndhD*, the other variable hotspot regions, *rpl32-trnL-UAG*, *rbcL-psaI*, and *rps18-rpl20*, are different from those in *S. japonicum* [50]. Furthermore, none of the 12 highly variable non-coding regions and 9 highly variable coding regions in *Utricularia amethystine* are the same as those in *S. japonicum* [56]. These variable hotspot regions, especially the highly variable sites in non-coding regions, can provide rich genetic information and are commonly used to analyze phylogenetic relationships between species and explore plant evolution. However, the taxon specificity of highly variable regions requires us to identify the specific variable hotspot regions unique to the target taxon and then develop highly variable molecular markers.

### 4.3. Intraspecific Phylogenetic Relationship of S. japonicum

Sun et al. [21] first used SRAP markers to conduct UPGMA analysis on some varieties of *S. japonicum*, exploring the genetic relationships between these varieties. The range of similarity coefficients detected among the samples was 0.68 to 0.89, with an average of 0.785, indicating high genetic similarity, small variability, and narrow genetic basis among the varieties, which is consistent with our results from chloroplast genome research. In Sun et al.’s clustering analysis, they observed the following: *S. japonicum* f. *oligophylla* formed a separate branch; *S. japonicum* f. *pendula* and *S. japonicum* ‘Flavi-rameus’ clustered together; *S. japonicum* ‘Shuangjimi’, *S. japonicum* ‘Winter Gold’, and *S. japonicum* ‘Liaohong’ clustered as another branch. This is different from our results based on complete chloroplast genome sequences. In our phylogenetic tree, *S. japonicum* ‘Shuangjimi’ and *S. japonicum* f. *pendula* clustered together, and *S. japonicum* ‘Liaohong’, *S. japonicum* ‘Winter Gold’, and *S. japonicum* ‘Flavi-rameus’ clustered together. The reason for these differences may be due to different genetic patterns of molecular markers, as chloroplast genomes are maternally inherited while SRAP markers are biparentally inherited. In both studies based on genetic variation, *S. japonicum* f. *oligophylla* was separated from other varieties and formed a separate branch, indicating different genetic origins from other varieties. Morphologically, especially in the compound leaf structure, *S. japonicum* f. *oligophylla* also showed significant differences from other varieties.

In the systematics of the chloroplast genome, eight major cultivars of *S. japonicum* are divided into two groups. Among them, only the cultivar *S. japonicum* ‘JinhuaiJ2’ forms a branch with two variants and one forma, while the remaining seven cultivars and one forma of *S. japonicum* f. *pendula* form another major branch, revealing that the main cultivars of *S. japonicum* may have two domestication sources. The phenotypic differences among the cultivated varieties of *S. japonicum* are mainly concentrated in branch color, branch morphology, leaf color, and fruiting characteristics. The main morphological feature of *S. japonicum* f. *pendula* is twisted branches, soft and drooping twigs, and an umbrella-shaped crown, mainly used for landscaping and an excellent tree species for greening [57]. *S. japonicum* ‘Shuangjimi’ blooms twice a year and is an excellent Huaimi crop cultivar [58]. The leaves of the cultivar *S. japonicum* ‘Flavi-rameus’ are golden yellow and belong to the category of ornamental foliage trees [59]. Our research shows that diverse cultivars of *S. japonicum* have close genetic relationships, which lays a theoretical foundation for the subsequent breeding of high-quality cultivars.

## 5. Conclusions

In this study, we provide a comprehensive description of the architecture of the *S. japonicum* cp genome, including its basic features, repeat sequences, SSRs, and phylogenetic relationships. Furthermore, we compare the cp genomes of different varieties of *S. japonicum*. The cp genome of *S. japonicum* exhibits a typical quadripartite structure, with 129 annotated functional genes, including 83 protein-coding genes, 38 tRNA genes, 8 rRNA genes, and 1 pseudogene. We found that the genetic diversity within *S. japonicum* chloroplast genomes was relatively low. Additionally, we identified eight highly variable regions as potential molecular markers for *Styphnolobium* species, which could be utilized in population genetic studies. The constructed phylogenetic tree revealed that the major cultivars of *S. japonicum* originated from two distinct genetic sources. These findings enhance our understanding of cp genomics and the genetic diversity of *S. japonicum*, providing a solid foundation for future research on molecular marker development, phylogenetic analysis, population studies, and cp genome engineering.

## Figures and Tables

**Table 1:** Comparison of the chloroplast genome features among twelve *S. japonicum* samples (including three sequences from NCBI).

Species Names	GenBank	Size/bp				No. of Genes				GC Content%			
		Genome	LSC	SSC	IR	Genome	PCG	tRNA	rRNA	Genome	LSC	SSC	IR
*S. japonicum* var. *japonicum*	MG784459	158,738	89,003	19,057	50,678	129	83	38	8	36.1%	33.5	29.61	43.15
*S. Japonicum* var. *violacea*	KY872756	158,837	88,983	19,058	50,796	129	83	38	8	36.1%	33.51	29.61	43.17
*S. japonicum f. pendula*	ON571617	158,645	88,905	19,060	50,680	129	83	38	8	36.1%	33.53	29.6	43.15
*S. japonicum f. oligophylla*	ON571618	158,739	89,003	19,058	50,678	129	83	38	8	36.1%	33.5	29.61	43.15
*S. Japonicum* ‘Jinyechui’	ON571616	158,613	88,871	19,062	50,680	129	83	38	8	36.1%	33.54	29.6	43.15
*S. japonicum* ‘Flavi-rameus’	N571615	158,616	88,868	19,068	50,680	129	83	38	8	36.1%	33.54	29.59	43.15
*S. Japonicum* ‘Winter Gold’	ON571614	158,624	88,876	19,068	50,680	129	83	38	8	36.1%	33.54	29.59	43.15
*S. japonicum* ‘Liaohong’	ON571620	158,646	88,897	19,069	50,680	129	83	38	8	36.1%	33.53	29.59	43.15
*S. japonicum* ‘Pingding’	ON571621	158,613	88,873	19,062	50,678	129	83	38	8	36.1%	33.54	29.6	43.15
*S. japonicum* ‘Susheng’	ON553276	158,629	88,876	19,073	50,680	129	83	38	8	36.1%	33.54	29.58	43.15
*S. japonicum* ‘Shuangjimi’	ON571619	158,639	88,907	19,054	50,678	129	83	38	8	36.1%	33.53	29.61	43.15
*S. japonicum* ‘JinhuaiJ2’	MN701078	158,690	88,977	19,034	50,680	129	83	38	8	36.1%	33.51	29.65	43.15

**Table 2:** List of genes in the chloroplast genome of *S. japonicum*.

Category	Gene Group	Gene Name
Photosynthesis	Subunits of photosystem I	*psaA*, *psaB*, *psaC*, *psaI*, *psaJ*
	Subunits of photosystem II	*psbA*, *psbB*, *psbC*, *psbD*, *psbE*, *psbF*, *psbH*, *psbI*, *psbJ*, *psbK*, *psbL*, *psbM*, *psbN*, *psbT*, *psbZ*
	Subunits of NADH dehydrogenase	*ndhA* *, *ndhB* *(2), *ndhC*, *ndhD*, *ndhE*, *ndhF*, *ndhG*, *ndhH*, *ndhI*, *ndhJ*, *ndhK*
	Subunits of cytochrome b/f complex	*petA*, *petB* *, *petD* *, *petG*, *petL*, *petN*
	Subunits of ATP synthase	*atpA*, *atpB*, *atpE*, *atpF* *, *atpH*, *atpI*
	Large subunit of rubisco	*rbcL*
Self-replication	Proteins of large ribosomal subunit	*rpl14*, *rpl16* *, *rpl2* *(2), *rpl20*, *rpl23 (2)*, *rpl33*, *rpl36*
	Proteins of small ribosomal subunit	*rps11*, *rps12* *(2), *rps14*, *rps15*, *rps16* *, *rps18*, *rps19*, *rps2*, *rps3*, *rps4*, *rps7 (2)*, *rps8*
	Subunits of RNA polymerase	*rpoA*, *rpoB*, *rpoC1* *, *rpoC2*
	Ribosomal RNAs	*rrn16S (2)*, *rrn23S (2)*, *rrn4.5S (2)*, *rrn5S (2)*
	Transfer RNAs	*trnA-UGC* *(2), *trnC-GCA*, *trnD-GUC*, *trnE-UUC*, *trnF-GAA*, *trnG-GCC*, *trnH-GUG*, *trnI-CAU (2)*, *trnI-GAU* *(2), *trnK-UUU* *, *trnL-CAA (2)*, *trnL-UAA* *, *trnL-UAG*, *trnM-CAU*, *trnN-GUU (2)*, *trnP-GGG*, *trnP-UGG*, *trnQ-UUG*, *trnR-ACG (2)*, *trnR-UCU*, *trnS-GCU*, *trnS-GGA*, *trnS-UGA*, *trnT-CGU* *, *trnT-GGU*, *trnT-UGU*, *trnV-GAC(2)*, *trnV-UAC* *, *trnW-CCA*, *trnY-GUA*, *trnfM-CAU*
Other genes	Maturase	*matK*
	Protease	*clpP* **
	Envelope membrane protein	*cemA*
	Acetyl-CoA carboxylase	*accD*
	c-type cytochrome synthesis gene	*ccsA*
Genes of unknown function	Conserved hypothetical chloroplast ORF	*ycf1 (2)*, *ycf2 (2)*, *ycf3* **, *ycf4*

Notes: Gene *: Gene with one intron; Gene **: Gene with two introns; Gene (2): Number of copies of multi-copy genes.

**Table 3:** Nucleotide diversity (Pi, Theta-W) of twelve *S. japonicum* samples.

	Genome	LSC	IRa	SSC	Noncoding Region	Coding Region
Total number of sites	159,102	89,215	25,399	19,089	81,133	78,072
Number of polymorphic sites	132	59	18	38	87	45
Pi	0.00029	0.00026	0.00014	0.00085	0.00043	0.00019
Theta-W:	0.00028	0.00022	0.00024	0.00066	0.00043	0.00019

## Data Availability

The data that support the findings of this study are publicly available in the National Center for Biotechnology Information (NCBI) at https://www.ncbi.nlm.nih.gov, accession number ON571614, ON571621 and ON553276.

## Supplementary Materials

The following supporting information can be downloaded at: https://www.mdpi.com/article/10.3390/genes14061156/s1, Figure S1: Variable characters in homologous regions among *S. japonicum* samples; Figure S2: Collinear analysis of the chloroplast genome of twelve *S. japonicum* samples.
