# Supplementary figures and images for: Intraspecific Chloroplast Genome Variation and Domestication Origins of Major Cultivars of Styphnolobium japonicum
Source: Genes (Basel). 2023 May 26;14(6):1156. doi: 10.3390/genes14061156 (PMC10297897; doi:10.3390/genes14061156)

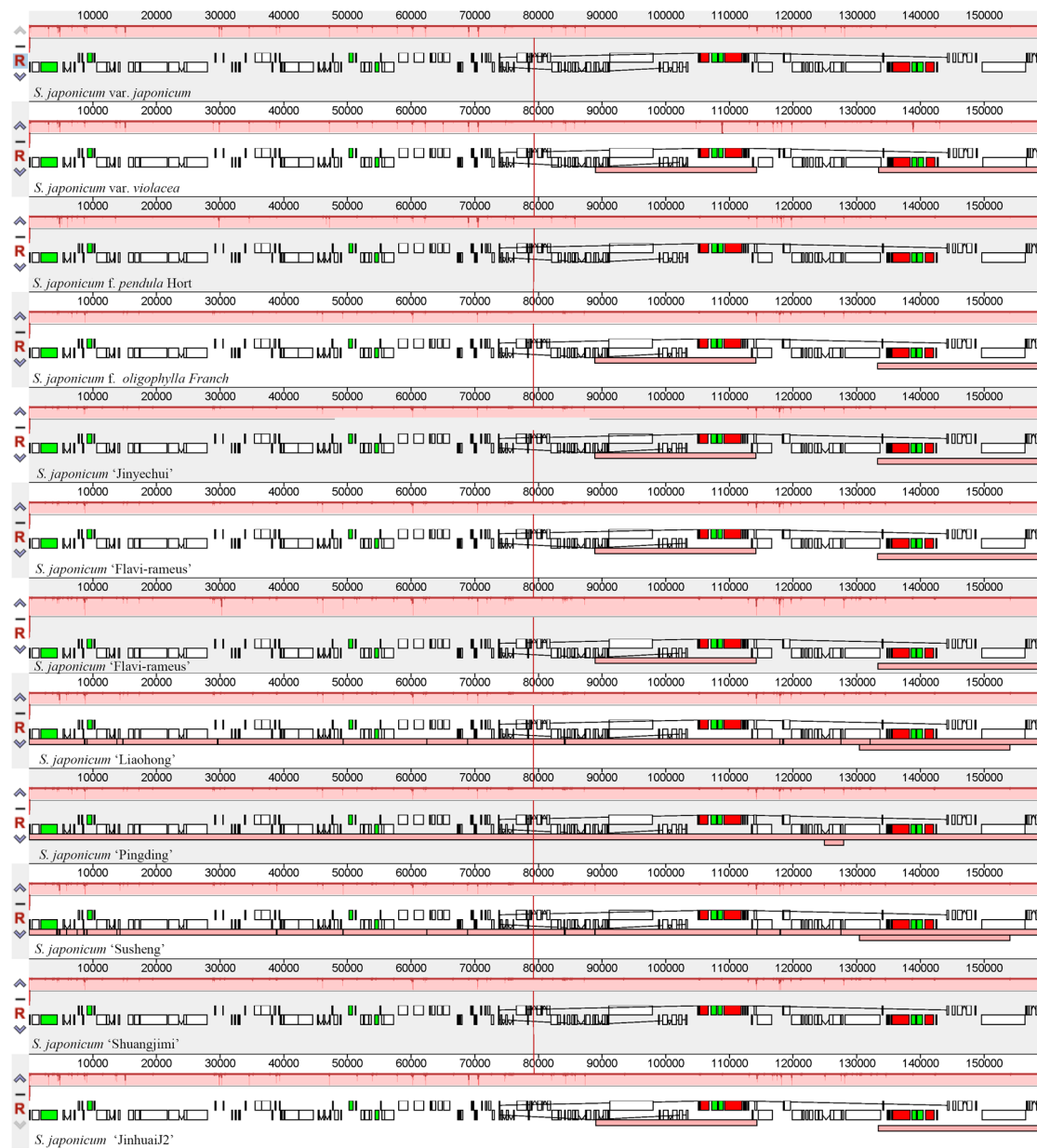

Figure S2: Collinear analysis of the chloroplast genome of twelve *S. japonicum* samples.

Supplement: Supplementary file 1 [file genes-14-01156-s001.zip › genes-2377225-supplementary.pdf]
